# A Rare Case of Hypercalcemia from Mediastinal Ectopic Hyperparathyroidism

**DOI:** 10.3390/jcdd12060201

**Published:** 2025-05-28

**Authors:** Nasrin Dhapa, Lamar Alocozy, Rumana Khan

**Affiliations:** Sutter Roseville Medical Center, Graduate Medical Education, Roseville, CA 95661, USA; lamar.alocozy@sutterhealth.org (L.A.); rumana.khan@sutterhealth.org (R.K.)

**Keywords:** primary hyperparathyroidism, hypercalcemia, ectopic disease, endocrinology

## Abstract

Hypercalcemia is frequently attributed to primary hyperparathyroidism, commonly a result of parathyroid adenomas. Ectopic hyperparathyroidism is characterized by hyperfunctioning parathyroid tissue located outside of expected anatomical locations of endocrine tissue. In this report, we present a rare case of hypercalcemia secondary to a mediastinal ectopic parathyroid adenoma, located between the left atrium and pulmonary artery. Given the unique location of the ectopic gland, diagnosis was delayed with additional complications that followed due to difficulty accessing the gland surgically. Despite this, urgent surgical removal of the ectopic gland allowed for remarkable improvement in presenting symptoms. This clinical case highlights diagnostic and therapeutic challenges that present a unique situation worthy of clinical discussion.

## 1. Introduction

Hypercalcemia is commonly associated with primary etiologies most commonly seen with parathyroid gland adenomas. However, ectopic parathyroid adenomas remain a challenge to diagnose. The rarity of ectopic parathyroid glands may result from aberrant migration during stages of development. One theory shares how orthotopic glands like adenomas may become larger and heavier leading to displacement, while another theory supports the abnormal migration of parathyroid tissue during embryogenesis leading to the atypical location [1]. The prevalence of these glands can be up to 16% in patients with primary hyperparathyroidism, and 14% in those with secondary hyperparathyroidism [2]. However, of these, a small constitute, about 10–15%, are found in the mediastinum surrounded by vital organs.

Mendoza and colleagues conducted a retrospective study of cases of primary hyperparathyroidism (PHPT) to assess the prevalence of ectopic and orthotopic parathyroid glands. Over a five-year period, 145 cases of PHPT were treated surgically. Of these, an ectopic parathyroid location was identified in 13 cases, or 9% of the time. Four were at the tracheoesophageal groove, four were intrathymic, two were intrathyroidal, and one each at the aortopulmonary window, anterior mediastinum, and submaxillary region. Patients with PHPT from ectopic glands have also been found to have significantly higher calcium levels than those with orthotopic parathyroid glands when symptomatic [3].

To add to the complexity, many ectopic parathyroid glands can be silent with no initial signs or symptoms presented; however, these can be unmasked in certain physiological states such as illness. In fact, the most common cause of persistent primary hyperparathyroidism is the presence of an ectopic gland that was not identified previously [1]. Parathyroid glands are commonly imaged with the use of CT or ultrasound. However, as ectopic glands are not readily visible, sestamibi scans are frequently utilized given their high sensitivity and specificity for parathyroid adenomas [2]. These scans utilize sestamibi molecules that are not taken up by orthotopic parathyroid glands; rather, they are rapidly absorbed by overactive glands due to the high affinity of these cells to the sestamibi. The use of these scans, although helpful in complex clinical scenarios, is controversial as false negative scans, affected by gland size, pathological variants, or multiglandular disease, can affect surgical outcomes [4]. Interestingly, the more recent use of 11-C Methionine and 18F-Fluorocholine PET studies appear to be promising second-line imaging modalities. In one study, the diagnostic accuracy of these studies was compared and the results suggested increased sensitivity with the use of 18F-Fluorocholine. However, it is important to note that these imaging types have been more commonly used in the diagnosis of primary hyperparathyroidism and their utility with the diagnosis of ectopic glands is unknown [5].

Given their occasional asymptomatic nature, surgery may not always be recommended. In fact, without preoperative localization, which in many cases can be difficult, 33–40% of cases are unable to identify the glands despite open sternotomy [6]. Surgical indications can include urine calcium > 10 mmol/24 h, signs of renal failure with a greater than 30% reduction in creatinine clearance, a T score greater than −2.5 on a bone density scan, or an adjusted calcium over 0.25 mmol/L. An alternate source also shares additional indications for intervention, which includes nephrolithiasis or even an age < 50 years alone, which can be sufficient to justify surgical intervention [7].

In this report, we highlight a unique case of hypercalcemia caused by a mediastinal ectopic parathyroid adenoma discovered between the left atrium and pulmonary artery.

## 2. Case Presentation

A sixty-four-year-old male with a past medical history of gastroesophageal reflux disease, prior methamphetamine abuse, now sober x 3 years, and 40-pack year smoking history presented to the emergency department after outpatient lab work demonstrated elevated calcium. The patient was first notified of hypercalcemia seven months prior when testing revealed a calcium level of 11.6 mg/dL. He was advised to follow up for repeat blood work in a few months and increased hydration. During this time, the patient began to develop significant polyuria but had no evidence of flank pain, kidney stones, bone pain, or constipation. Complete metabolic panel on the day of the emergency department visit was notable for a calcium level of 13.0 mg/dLwith an elevated parathyroid hormone of 404 pg/mL. The patient was initiated on judicious intravenous fluids and admitted for further workup.

Given the significant smoking history, a computed tomography (CT) scan of the chest and parathyroid-related hormone-related peptide was completed the following morning. At this point, the top differential remained primary hyperparathyroidism, supported by a parathyroid ultrasound demonstrating a left-sided adenoma, further supporting this. The calcium level slowly improved with intravenous fluids; however, given the continued elevation in calcium, cinacalcet was also initiated. A renal ultrasound resulted in negative findings for nephrolithiasis. Despite the continuation of IV fluids and cinacalcet, the patient’s calcium remained elevated, ranging from 12.5 to 13.3 mg/dL. In addition, acute kidney injury developed as a result of the polyuria from hypercalcemia. A curbside consultation with outpatient endocrinology was completed with recommendations for urgent outpatient follow-up with an endocrine specialist. The patient was discharged on cinacalcet with a calcium level of 12.9 mg/dL. A repeat renal panel was also ordered to be completed within two days of discharge for continued monitoring.

Two days after discharge, the patient completed outpatient lab work which revealed a rise in calcium to 14.0 mg/dL, leading to his subsequent presentation to the ED. Given the persistence of hypercalcemia, an urgent otolaryngology (ENT) referral was obtained for inpatient surgical evaluation. The ENT specialist recommended a sestamibi scan to confirm if the lesion on the ultrasound was hyperfunctioning. Pending these results, there would be a tentative plan for parathyroid exploration and an excision of the left lower parathyroid gland where the suspected adenoma was identified on ultrasound. Interestingly, however, the nuclear medicine scan noted unusual findings of uptake in the mediastinum, documented as radiotracer avid soft tissue density immediately superior to the left atrium, interposed between the right main pulmonary artery and left atrium, measuring approximately 24 × 23 mm—all in all, a highly unusual location for parathyroid tissue.

**Figure 1:**
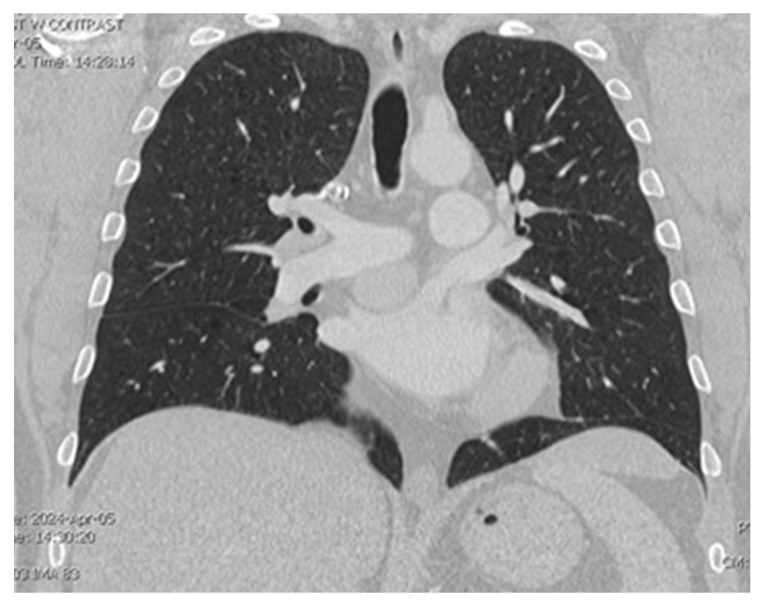
Visualization of the mass located between the pulmonary artery and left atrium on CT chest.

**Figure 2:**
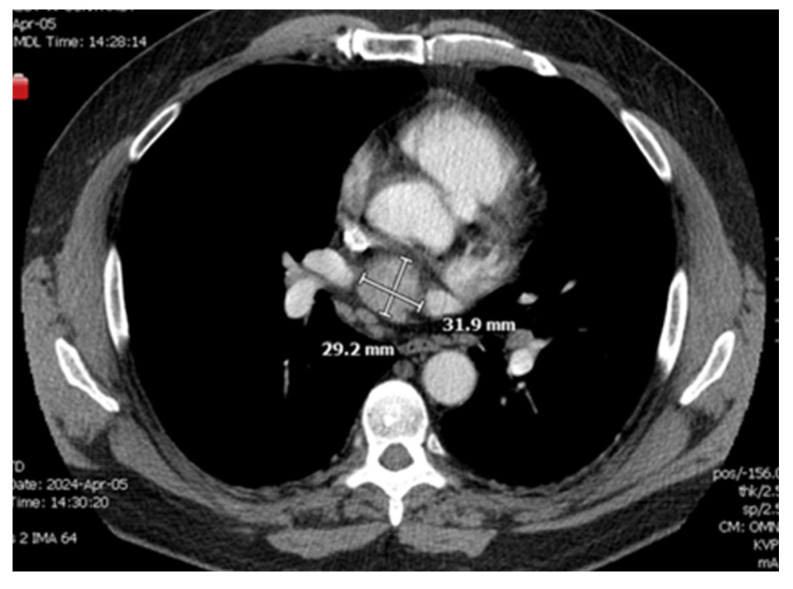
Re-demonstration of mediastinal mass in Axial CT scan.

A CT chest scan, now with contrast, was completed on 4/5 and demonstrated a solid mildly enhancing 3.2 cm mass located just superior to the left atrium and just inferior to the right pulmonary artery consistent with findings from the nuclear medicine scan (Figure 1 and Figure 2). There remained a concern for ectopic parathyroid tissue but also a malignant tumor, hence a recommendation was made for tissue sampling. Parathyroid exploration was deferred given that orthotopic parathyroid pathology did not appear to be the source. Thoracic surgery was consulted for possible surgical removal of the gland; however, there was concern about safe accessibility given the location of the gland.

The case was also discussed with interventional radiology for a possible CT-guided biopsy, but the procedure was again deemed unsafe. Similarly, the pulmonology team was consulted for an endobronchial ultrasound biopsy while the gastroenterology team was consulted for a possible endoscopic ultrasound guided biopsy. Although the procedure was declined by pulmonology, gastroenterology did note that although there was a narrow window for endoscopic ultrasound biopsy, it may be possible. Given the persistent hypercalcemia, the patient declined biopsy and requested definitive surgical intervention with the removal of the gland. Thoracic surgery was consulted a second time but due to the treacherous location of the mass, the patient was deemed to require multispecialty intervention to ensure safe outcomes. Hence, it was deemed that cardiac surgery expertise would also be required, and the patient was transferred to a local hospital with cardiothoracic service availability.

A cardiothoracic surgeon evaluated the patient with plans for surgical resection with an angiogram prior to open sternotomy in the case of any coronary disease that may similarly need intervention. The patient underwent mediastinal exploration while on cardiopulmonary bypass with the successful excision of the ectopic gland, noted to be a 3.5 × 2.8 cm solid mass sent for pathology.

Postoperatively, the patient was monitored in the cardiac ICU with subsequent lab-work demonstrating improvement in calcium levels and the resolution of elevated parathyroid hormone, initially 690 pg/mL now improved to 305 pg/mL and later 198 pg/mL. Pathology results demonstrated a hypercellular parathyroid gland. A week after surgery, the patient was evaluated in an outpatient clinic and noted significant improvement in his symptoms. Approximately one month later, the patient was noted to be walking 10–15 min daily with the resolution of his polyuria

## 3. Discussion

Not only are ectopic parathyroid glands a challenge to diagnosis, but the location of these glands can also further complicate the clinical picture. In this case, the patient did not have severe symptoms on presentation, leading to invasive workup being deferred, further delaying the identification of the gland.

One prior case discusses a 75-year-old male who was presented to an endocrinology clinic after chronic hypercalcemia was noted in lab work. Other than intermittent abdominal pain, the patient was asymptomatic. Lab work demonstrated a calcium level of 12 mg/dL with elevated parathyroid hormone. Twenty-four-hour urine calcium collection was noted to be 16.77 mmol/L in the twenty-four-hour period. Initial ultrasound demonstrated a small hypoechoic soft tissue nodule adjacent to the inferior pole of the right thyroid. A follow up computer tomography scan demonstrated a well-defined nodule involving the left side of mediastinum anterior to aortic arch and posterior to manubrium. To further confirm diagnosis, a sestamibi scan was performed showing distinct focus at the left side of the anterior mediastinal region, highly suggestive of ectopic parathyroid adenoma. The patient met indications for surgical intervention and underwent a left thoracoscopic mediastinal parathyroidectomy. Postoperatively, the PTH level improved, calcium down trended, and the patient was discharged 3 days later [8]. This case and the one we report demonstrated that mediastinal adenomas can be extremely difficult to identify. Even after these glands are better localized through nuclear scans, surgical challenges persist given the proximity to cardiopulmonary structures [9].

Although the patient discussed in the above case report and this article had clear indications for surgical intervention, concurrent medical management remains critical. Hypercalcemia frequently leads to volume depletion; hence, intravenous hydration is critical, typically with isotonic saline. Additionally, electrolyte monitoring and repletion is important to maintain normal levels. Calcitonin can be administered as an intramuscular or subcutaneous injection to lower calcium levels quickly, working as quickly as 2 h after onset. However, the effects of this medication are limited and may only last 4–7 days [10]. Bisphosphonates, like pamidronate and zoledronic acid, are approved for hypercalcemia treatment; however, these medications do take up to 72 h to demonstrate effect and hence, are more commonly utilized along with the other methods noted above. Cinacalcet is a calcium mimetic and can also be commonly applied in secondary hyperparathyroidism due to renal failure [11].

While many mediastinal adenomas are resected via cervical invasion, this can be difficult depending on where the adenoma is located relative to the aortic arch. One study suggests approaching adenomas found above the aortic arch transcervically, while those in the middle or posterior mediastinum below the aortic arch may better be approached through the transthoracic approach [9]. Additionally, using a gamma probe intraoperatively can further help identify the gland and reduce operating time, although this does increase the risk of radioisotopes in the myocardium [12]. It is also crucial to discuss options for patients who may not be surgical candidates. In these situations, angiographic ablation can be utilized by injecting extra contrast material into the supplying vessel, resulting in ischemic change to the gland [13].

## 4. Conclusions

The presented case underscores the diagnostic challenges and therapeutic successes in managing hyperparathyroidism when caused by ectopic tissue situated in between critical organs. The meticulous preoperative localization using advanced imaging techniques facilitated precise surgical planning, leading to the successful removal of the ectopic gland and resolution of the patient’s symptoms. This case highlights the importance of thorough investigation and individualized management in achieving favorable outcomes for patients who may otherwise present with benign medical problems. Innovative approaches remain crucial in navigating complex cases, ensuring optimal patient care and long-term well-being.

## Data Availability

Data are available on request due to restrictions.
